# Common genetic variants in pulmonary arterial hypertension

**DOI:** 10.1016/S2213-2600(18)30448-X

**Published:** 2018-12-05

**Authors:** Sue Gu, Rahul Kumar, Michael H Lee, Claudia Mickael, Brian B Graham

**Affiliations:** Program in Translational Lung Research, Department of Medicine, University of Colorado Anschutz Medical Campus, Aurora, CO 80045, USA

Pulmonary arterial hypertension is currently an incurable disease characterised by increased pulmonary vascular resistance and eventual death due to right ventricular failure. Hereditary pulmonary arterial hypertension has been generally thought to represent a small subset of all pulmonary arterial hypertension cases. The most common mutation identified is loss of function in *BMPR2*, which encodes a cell surface receptor belonging to the transforming growth factor-β (TGFβ) superfamily.^1,2^ Understanding of the pathogenetic role of *BMPR2* haploinsufficiency has uncovered new potential therapeutic targets in pulmonary arterial hypertension, with multiple clinical trials focusing on the TGFβ signalling pathway. Despite the documented contribution of common genetic variants as determinants of complex and multifactorial diseases, previous genome-wide association studies (GWAS) in patients with pulmonary arterial hypertension disease have been scarce. In *The Lancet Respiratory Medicine*, Christopher Rhodes and colleagues^3^ report their findings from the largest genetic analysis to date in patients of European ancestry with pulmonary arterial hypertension. By use of two independent GWAS platforms of whole-genome sequencing and genotyping array (discovery phase), and meta-analysis (validation phase) with multiple internationally collaborative cohorts, the study identified two novel loci associated with risk for the development of pulmonary arterial hypertension: an enhancer region in *SOX17*, and a locus within *HLA-DPA1* and *HLA-DPB1*. The alleles associated with disease were common, with a relatively modest effect size: for example, 59% of patients with pulmonary arterial hypertension compared with 46% of controls were homozygous for the risk allele at both *SOX17* SNPs. The results of the discovery and validation phases converged to substantially decrease the possibility of false-positive results.

The authors reported two independent signals in the transcription factor *SOX17* locus significantly associated with pulmonary arterial hypertension (rs10103692, odds ratio 1.80 [95% CI 1.55–2.08], p=5.13 × 10^−15^, and rs13266183, 1.36 [1.25–1.48], p 1.69 × 10^−12^). These signals identified enhancer regions leading to modified expression of *SOX17*, which correlated with the diagnosis of pulmonary arterial hypertension. The functional impact of the novel *SOX17* locus on pulmonary arterial hypertension susceptibility was confirmed with Hi-C to determine DNA folding patterns and CRISPR-mediated inhibition in human pulmonary artery endothelial cells. This finding corroborates a previous report describing a rare genetic variant in *SOX17* associated with heritable pulmonary arterial hypertension.^4^ The present study posits that common variation in *SOX17* expression is a determinant of pulmonary arterial hypertension and is present more often in patients with pulmonary arterial hypertension than are other rare genetic variants. Conditional deletions of *SOX17* in endothelial cells cause abnormal pulmonary vascular morphogenesis^5^ potentially mediated by Notch pathway signalling to restrict angiogenesis.^6^ Inactivation of *SOX17* in mouse embryos leads to lack of arterial differentiation and vascular remodelling,^7^ and SOX17 has a major role in the development of haemogenic endothelial cells (the precursor of haemopoietic stem cells).^8^

The other key genes identified by this study were *HLA-DPA1* and *HLA-DPB1* (rs2856830, 1.56 [1.42–1.71], p=7.65 × 10^−20^), which encode the MHC class II DP α and β chains. The specific variant discovered was located in *HLA-DPB1* and, surprisingly, correlated with two seemingly opposite outcomes: increased risk for the development of pulmonary arterial hypertension, but improved survival once having developed pulmonary arterial hypertension. The specific alleles associated with the variants were HLA-DPB1*02:01/02:02/16:01, all of which contain a glutamic acid substituted for a lysine residue at position 69. Of note, variants with the same glutamic acid substitution at position 69 have also been described as increasing the risk for developing chronic beryllium disease (a variant of sarcoidosis) among individuals exposed to beryllium.^9^ In studies using crystallography, the negative charge of the glutamine side chain was found to stabilise the Be^2+^ ion in the MHC II groove, facilitating a neoantigen that triggers a granulomatous lung disease.^10^ The finding of the same variants in these patients raises the possibility of an antigenic trigger contributing to the development of pulmonary arterial hypertension. This antigen-triggered disease might have a more benign disease course or improved clinical response to the current clinical armamentarium compared with other forms of pulmonary arterial hypertension, resulting in the improved survival observed.

On the basis of the current findings, the next steps include first reproducing and validating these findings with additional and more diverse samples. It will then be important to associate the risk alleles of *SOX17* and *HLA-DPB1* with clinical characteristics and relevant pathologies in pulmonary arterial hypertension, including inflammatory cytokines, incidence of vasodilator responsiveness, haemodynamics, and right ventricle performance, which will help to contextualise the relevance of the GWAS data to the clinical setting. It remains unanswered whether the significance of these markers generalises to ethnicities, whether these markers drive similar clinical presentations and outcomes in both sexes, whether they can serve as biomarkers to determine predisposition towards and stratification of pulmonary arterial hypertension subtypes, or whether they interact with other genes in a complex disease such as pulmonary arterial hypertension. Transgenic modification of experimental animals with the same mutations will further clarify whether and how these modifications induce disease and provide insights into how they can be pharmacologically targeted. Overall, this study provides hope that the identification of genetic modifiers in pulmonary arterial hypertension will allow more accurate classification of pulmonary arterial hypertension subtypes and more tailored and effective treatments for patients with pulmonary arterial hypertension than are currently available.
